# Prevalence and Predictors of Post-coital Hypoglycemia in Diabetes Mellitus

**DOI:** 10.7759/cureus.91290

**Published:** 2025-08-30

**Authors:** Qasem Al Jabr, Ali A Bu-Khamseen, Mohammed Alalawi, Ali A Alkuwaiti, Baqer A Aldhneen, Fatimah J Al-Shehab, Hassan Al Ameer, Fatimah Al Dakhlan, Norah Albaqshi, Alya Bukhamsin

**Affiliations:** 1 Family Medicine, Geriatric Medicine, Postgraduate Center of Family Medicine in Al-Ahsa, Al-Ahsa, SAU; 2 Family Medicine, Al-Ahsa Health Cluster, Mubarraz, SAU; 3 Family Medicine, Al-Ahsa Family Medicine Academy, Al-Ahsa, SAU; 4 Familly Medicine, Al-Ahsa Health Cluster, Al-Ahsa, SAU; 5 Family Medicine, Al-Ahsa Health Cluster, Al-Ahsa, SAU; 6 Emergency, Al-Ahsa Health Cluster, Al-Ahsa, SAU; 7 Familly Medicine, King Faisal University, Al-Ahsa, SAU; 8 Public Health, King Faisal University, Al-Ahsa, SAU

**Keywords:** insulin therapy, post-coital hypoglycemia, sexual health, type 1 diabetes mellitus, type 2 diabetes mellitus

## Abstract

Post-coital hypoglycemia is a significant yet often overlooked complication in diabetes management. This cross-sectional study aimed to assess the prevalence and associated factors of post-coital hypoglycemia among patients with type 1 diabetes mellitus (T1DM) and type 2 diabetes mellitus (T2DM) in Al-Ahsa, Saudi Arabia. A total of 821 participants were interviewed via telephone using a structured questionnaire. The study revealed that 17.9% of participants experienced hypoglycemic events during or after sexual activity. Insulin use was strongly associated with post-coital hypoglycemia, with 28.3% of insulin users reporting such events compared to only 3.0% of non-insulin users (p<0.001). T1DM patients were significantly more likely to experience post-coital hypoglycemia than T2DM patients (92.5% vs 7.5%, p<0.001). Younger age groups, higher educational levels, and married status were also associated with increased incidence. Common symptoms reported included sweating (47.6%), palpitations (42.0%), and dizziness (37.3%). The use of certain oral diabetic medications, including metformin, sitagliptin, and gliclazide-sulfonylurea, was also associated with higher rates of post-coital hypoglycemia. These findings highlight the need for increased awareness and targeted interventions to address post-coital hypoglycemia in diabetes management. Healthcare providers should incorporate discussions about sexual health and hypoglycemia risk into routine diabetes care, particularly for high-risk groups such as insulin users and T1DM patients. Future research should focus on developing effective strategies to prevent and manage post-coital hypoglycemia, thereby improving the quality of life for individuals with diabetes.

## Introduction

Diabetes mellitus (DM) is a prevalent chronic disorder resulting from impaired insulin secretion, insulin action, or both, and it remains a major global health challenge due to its high prevalence and burden of complications [1-3]. Type 1 diabetes mellitus (T1DM) is characterized by autoimmune destruction of pancreatic β-cells and absolute insulin deficiency, while type 2 diabetes mellitus (T2DM) is defined primarily by insulin resistance with relative insulin deficiency [4-10]. Despite differences in pathophysiology, both forms of diabetes require ongoing management to prevent acute and chronic complications [11].

Among these complications, hypoglycemia is one of the most frequent and burdensome acute events, negatively affecting quality of life and increasing morbidity and mortality [11,12]. Symptoms range from autonomic activation (sweating, palpitations, dizziness) to neuroglycopenic manifestations (confusion, seizures, loss of consciousness) [12]. Hypoglycemia risk is heightened in individuals using insulin or insulin secretagogues, particularly in situations of mismatched insulin action and energy expenditure [13-15].

Sexual activity represents a unique and understudied trigger for hypoglycemia. Physiological studies show that intercourse involves energy expenditure comparable to moderate-intensity exercise, especially in men, with caloric costs similar to treadmill-based activity [13,14]. Like exercise, sexual activity stimulates autonomic responses - sympathetic activation during arousal and parasympathetic shifts during recovery - that can increase glucose utilization and destabilize glycemic balance in insulin-treated patients [15-17]. Consequently, “post-coital hypoglycemia” can be conceptualized as a subset of exertion-related hypoglycemia, yet it remains poorly characterized in diabetes care [16-19].

Prior evidence indicates that many patients with T1DM do not implement preventive measures against hypoglycemia before sexual activity, leading to significant distress, impaired sexual well-being, and relationship strain [20,21]. A study by Geddes et al. [20] reported that 40% of adults with T1DM experienced hypoglycemic symptoms during or after intercourse, while others have highlighted the psychological burden of fear of hypoglycemia on sexual health [21]. Despite these findings, post-coital hypoglycemia remains largely absent from clinical discussions, educational programs, and research agendas.

Given this gap, the present study aims to assess the prevalence and predictors of post-coital hypoglycemia among patients with T1DM and T2DM in Al-Ahsa, Saudi Arabia. By examining demographic, clinical, and behavioral factors associated with this complication, the study seeks to generate evidence that can guide targeted counseling, risk mitigation, and patient education strategies, ultimately improving quality of life and sexual health for individuals with diabetes.

Aim of the study

The primary aim of this study is to assess the prevalence and associated factors of post-coital hypoglycemia in patients with T1DM and T2DM living in Al-Ahsa. By identifying the extent of this issue and understanding the demographic, clinical, and behavioral factors associated with post-coital hypoglycemia, this research seeks to provide insights that can lead to better management and preventive strategies, ultimately improving the quality of life for individuals with diabetes.

Research question

What is the prevalence of post-coital hypoglycemic events among patients with T1DM and T2DM living in Al-Ahsa, and what demographic, clinical, and behavioral factors are associated with these hypoglycemic events?

## Materials and methods

Study design

This study employed a cross-sectional design to assess the prevalence and predictors of post-coital hypoglycemia among patients with T1DM and T2DM in Al-Ahsa, Saudi Arabia. This design enabled estimation of prevalence and associated factors at a single point in time.

Study area and population

The study was conducted in Al-Ahsa, a large region in the Eastern Province of Saudi Arabia. Participants were adults aged 18 years and older with a confirmed diagnosis of T1DM or T2DM who resided in the region. Patients were identified through electronic health records from hospitals, diabetes clinics, and primary healthcare centers. Eligible participants were contacted by trained data collectors and invited to participate in structured telephone interviews.

Inclusion and exclusion criteria

Participants were included if they were adults with T1DM or T2DM and had experienced at least one hypoglycemic event confirmed by symptoms or by a self-monitored glucose value of 70 mg/dL or lower. Patients not diagnosed with T1DM or T2DM, those unable to engage in sexual activity, and non-residents of Al-Ahsa were excluded. Non-insulin users were excluded from the primary analysis to minimize misclassification bias, although we acknowledge that this decision may reduce generalizability and introduce selection bias.

Definition of post-coital hypoglycemia

Post-coital hypoglycemia was defined as a symptomatic or documented blood glucose value of 70 mg/dL or lower occurring during or within two hours after sexual activity, consistent with the American Diabetes Association definition of hypoglycemia.

Sample size and sampling

The sample size was estimated at 800 participants using Raosoft software, assuming a 5% margin of error, 95% confidence level, and a 50% response distribution. A convenience sampling approach was used, which facilitated access to a large and diverse cohort within the region, though it may limit external validity.

Data collection and questionnaire development

Data were collected between January 1, 2023, and December 30, 2023 using a structured telephone questionnaire. The tool covered demographic characteristics, diabetes history, general hypoglycemic events, and post-coital hypoglycemia experiences. It was developed following a review of relevant literature and reviewed by an expert panel of three endocrinologists and two diabetes nurse specialists to ensure content validity. To enhance clarity and cultural appropriateness, the questionnaire was pilot tested on 20 patients from the target population, and minor revisions were made before full administration. Trained data collectors conducted interviews after obtaining verbal informed consent, and participants were reassured of confidentiality to reduce underreporting of sensitive information.

Ethical considerations

Ethical approval was obtained from the King Fahad Hospital Ethics Committee (IRB Log No: 06-48-2022, H-05-HS-065). All participants provided verbal informed consent before enrollment. Data were anonymized and securely stored for at least three years in accordance with institutional ethical standards.

Statistical analysis

Data were analyzed using IBM SPSS Statistics version 26 (IBM Corp., Armonk, NY, USA). Descriptive statistics were used to summarize demographic and clinical characteristics. Associations between post-coital hypoglycemia and independent variables were examined using chi-square tests and t-tests. Variables significant in bivariate analyses were entered into multivariate logistic regression to identify independent predictors. Results were expressed as odds ratios with 95% confidence intervals, and a p-value less than 0.05 was considered statistically significant.

## Results

Table 1 presents the demographic and clinical characteristics of the study participants. The study comprised 821 participants with a balanced gender distribution, including 409 males (49.8%) and 412 females (50.2%). Participants primarily belonged to the 30-44-year age group (n=342, 41.7%), followed by the 45-59-year age group (n=298, 36.3%). Educational attainment varied, with the majority completing high school (n=236, 28.7%) or a bachelor’s degree (n=180, 21.9%). Most participants were married (n=762, 92.8%). Regarding diabetes type, 381 participants (46.4%) had T1DM, while 440 (53.6%) had T2DM. Over half of the participants (n=457, 55.7%) were diagnosed more than 10 years prior. Insulin was the most frequently used diabetic medication (n=484, 59.0%), followed by metformin (n=420, 51.2%). Additionally, beta blockers were used by 62 participants (7.6%).

**Table 1:** Demographic and Clinical Characteristics of Study Participants

Variable	Category	N	%
Gender	Male	409	49.8%
	Female	412	50.2%
Age group	15–29	104	12.7%
	30–44	342	41.7%
	45–59	298	36.3%
	60–74	77	9.4%
Educational level	Illiterate	63	7.7%
	Primary school	149	18.1%
	Intermediate	105	12.8%
	High school	236	28.7%
	Diploma	80	9.7%
	Bachelor	180	21.9%
	Postgraduate	8	1.0%
Marital status	Married	762	92.8%
	Single	27	3.3%
	Divorced	17	2.1%
	Widowed	15	1.8%
Type of diabetes	T1DM	381	46.4%
	T2DM	440	53.6%
When diagnosed	<6 years	212	25.8%
	6–10 years	152	18.5%
	>10 years	457	55.7%
Diabetic medications	Metformin	420	51.2%
	Sitagliptin	130	15.8%
	Insulin	484	59.0%
	Gliclazide-Sulfonylurea	100	12.2%
	Empagliflozin-SGLT2	57	6.9%
	GLP-1	20	2.4%
Beta blocker use	Yes	62	7.6%
	No	759	92.4%

Table 2 summarizes comorbidities among participants. Hypertension (HTN) was the most common comorbidity (n=239, 29.1%), followed by other unspecified conditions (n=97, 11.8%). Hypothyroidism affected 22 participants (2.7%), asthma 12 participants (1.5%), dyslipidemia six participants (0.7%), and chronic kidney disease (CKD) three participants (0.4%). Notably, 518 participants (63.1%) reported no comorbidities

**Table 2:** Comorbidities Among Study Participants

Comorbidity	n	%
Hypertension (HTN)	239	29.1%
Dyslipidemia	6	0.7%
Chronic Kidney Disease (CKD)	3	0.4%
Asthma	12	1.5%
Hypothyroidism	22	2.7%
Others	97	11.8%
Not Applicable	518	63.1%

**Figure 1:**
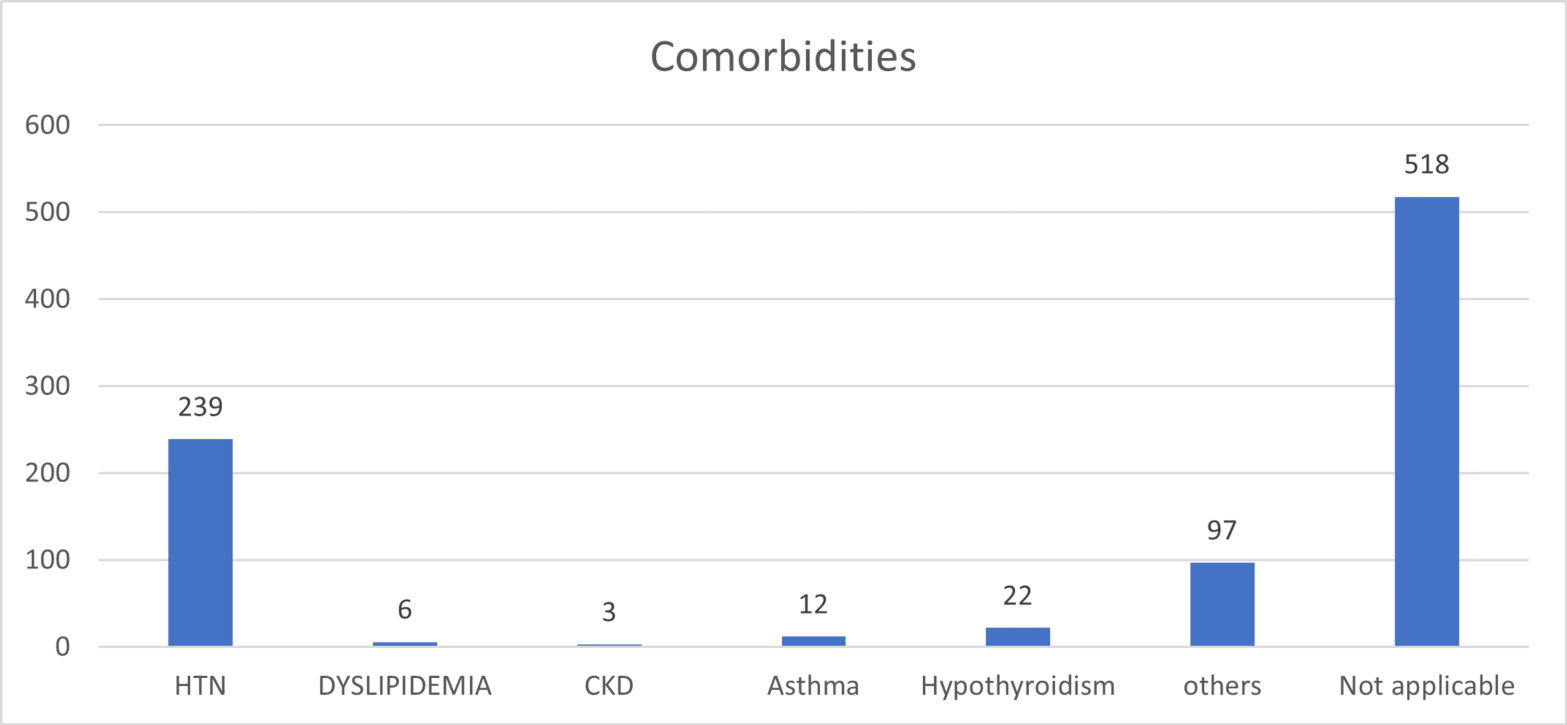
Distribution of Comorbidities Among Study Participants HTN: hypertension, CKD: chronic kidney disease

Figure 1 illustrates these findings, confirming that hypertension was the most prevalent comorbidity, affecting 29.1% of participants.

Table 3 shows that overall, 513 participants (62.5%) reported experiencing hypoglycemic episodes. Specifically, 147 participants (17.9%) reported experiencing hypoglycemia during or after sexual activity.

**Table 3:** Prevalence of Hypoglycemic Events Among Study Participants

Hypoglycemia Occurrence	n	%
In General		
No	308	37.5%
Yes	513	62.5%
During or After Coitus		
Yes	147	17.9%
No	514	62.6%
Not applicable	160	19.5%
Total	821	100.0%

Table 4 demonstrates a significant association between insulin use and the occurrence of hypoglycemic events during or after coitus. Among insulin users, 137 (28.3%) reported hypoglycemic events, compared to only 10 (3.0%) among non-insulin users (χ²=81.4, p<0.001).

**Table 4:** Association Between Insulin Use and Hypoglycemic Attacks During or After Coitus Data presented as N (%). Chi-square test used; χ² = 81.4, p < 0.001. A p-value < 0.05 was considered statistically significant.

Insulin Use	Hypoglycemic Attack (Yes) (n, %)	No Attack (n, %)	Not Applicable (n, %)	Total (n, %)	Chi-square (χ²)	p-value
No	10 (3.0%)	224 (66.5%)	103 (30.6%)	337 (100%)	81.4	<0.001
Yes	137 (28.3%)	290 (59.9%)	57 (11.8%)	484 (100%)	81.4	<0.001
Total	147 (17.9%)	514 (62.6%)	160 (19.5%)	821 (100%)	81.4	<0.001

Table 5 details associations between demographic variables and post-coital hypoglycemia. Gender showed no significant association (p=0.347). However, younger age groups (15-29 and 30-44 years) reported significantly higher incidences (χ²=88.3, p<0.001). Higher educational levels, particularly bachelor's degree holders, were significantly associated with post-coital hypoglycemia (χ²=52.7, p<0.001). Married status was also significantly associated with increased occurrence (χ²=78.5, p<0.001). Individuals with T1DM reported significantly higher rates of post-coital hypoglycemia compared to those with T2DM (92.5% vs. 7.5%; χ²=132.6, p<0.001).

**Table 5:** Association between Demographic Factors and Incidence of Hypoglycemic Attacks during or Post-Coitus

Demographic Factor	Category	Yes (n, %)	No (n, %)	Not Applicable (n, %)	Chi-square (χ²)	p-value
Gender	Male	66 (44.9%)	265 (51.6%)	78 (48.8%)	0.89	0.347
	Female	81 (55.1%)	249 (48.4%)	82 (51.3%)	0.89	0.347
Age group	15–29	36 (24.5%)	51 (9.9%)	17 (10.6%)	88.3	<0.001
	30–44	96 (65.3%)	204 (39.7%)	42 (26.3%)	88.3	<0.001
	45–59	12 (8.2%)	213 (41.4%)	73 (45.6%)	88.3	<0.001
	60–74	3 (2.0%)	46 (8.9%)	28 (17.5%)	88.3	<0.001
Educational level	Illiterate	1 (0.7%)	50 (9.7%)	12 (7.5%)	52.7	<0.001
	Primary	13 (8.8%)	95 (18.5%)	41 (25.6%)	52.7	<0.001
	Intermediate	18 (12.2%)	59 (11.5%)	28 (17.5%)	52.7	<0.001
	High school	42 (28.6%)	150 (29.2%)	44 (27.5%)	52.7	<0.001
	Diploma	24 (16.3%)	45 (8.8%)	11 (6.9%)	52.7	<0.001
	Bachelor	49 (33.3%)	108 (21.0%)	23 (14.4%)	52.7	<0.001
	Postgraduate	0 (0.0%)	7 (1.4%)	1 (0.6%)	52.7	<0.001
Marital status	Married	146 (99.3%)	496 (96.5%)	120 (75.0%)	78.5	<0.001
	Single	0 (0.0%)	7 (1.4%)	20 (12.5%)	78.5	<0.001
	Divorced	1 (0.7%)	10 (1.9%)	6 (3.8%)	78.5	<0.001
	Widowed	0 (0.0%)	1 (0.2%)	14 (8.8%)	78.5	<0.001
Type of diabetes	T1DM	136 (92.5%)	210 (40.9%)	35 (21.9%)	132.6	<0.001
	T2DM	11 (7.5%)	304 (59.1%)	125 (78.1%)	132.6	<0.001

Table 6 reveals a significant association between specific hypoglycemic symptoms and the incidence of post-coital hypoglycemia. Participants who experienced post-coital hypoglycemia reported higher rates of sweating (80.9%), palpitations (79.6%), dizziness (66.0%), confusion (25.2%), and hunger (45.6%) compared to those without such episodes (all p-values <0.001).

**Table 6:** Association Between Hypoglycemic Symptoms and Occurrence of Hypoglycemic Attack During or Post-Coitus (Data presented as N (%); Chi-square test applied; p < 0.05 considered statistically significant)

Symptom	Response	Hypoglycemia Yes (n, %)	Hypoglycemia No (n, %)	Not Applicable (n, %)	χ²	p-value
Sweating	Yes	119 (80.9%)	247 (48.5%)	25 (2.2%)	41.8	<0.001
Palpitation	Yes	117 (79.6%)	201 (40.4%)	27 (2.2%)	39.2	<0.001
Dizziness	Yes	97 (66.0%)	188 (37.8%)	21 (1.1%)	28.9	<0.001
Confusion	Yes	37 (25.2%)	84 (16.9%)	7 (0.7%)	9.3	<0.001
Feeling hungry	Yes	67 (45.6%)	117 (23.6%)	8 (0.8%)	23.7	<0.001
Others	Yes	130 (88.4%)	277 (55.5%)	36 (2.2%)	43.1	<0.001
NA (not reported)	Yes	1 (0.7%)	187 (36.4%)	118 (7.3%)	49.6	<0.001

Table 7 summarizes the prevalence of hypoglycemic symptoms among participants. Sweating (47.6%) was most common, followed by palpitations (42.0%), dizziness (37.3%), hunger (23.4%), and confusion (15.6%). Additionally, 54.0% reported experiencing other unspecified symptoms.

**Table 7:** Prevalence of Hypoglycemic Symptoms among Participants

Symptom Experienced	n	%
Sweating	391	47.6%
Palpitation	345	42.0%
Dizziness	306	37.3%
Confusion	128	15.6%
Feeling hungry	192	23.4%
Other symptoms reported	443	54.0%
Not Applicable (no symptoms reported)	306	37.3%

**Figure 2:**
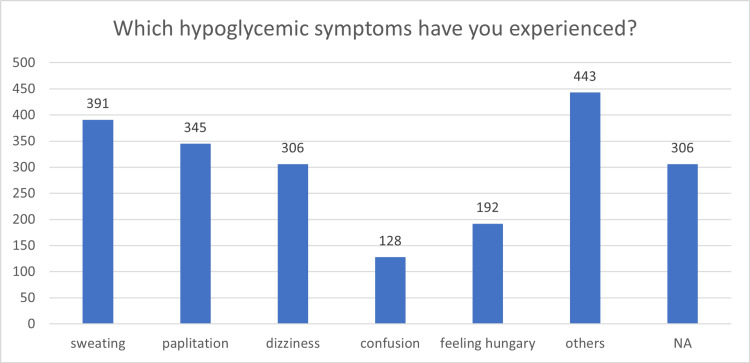
Hypoglycemic Symptoms Experienced by Study Participants

Figure 2 provides a graphical representation of these hypoglycemic symptoms, highlighting the predominance of sweating, palpitations, and dizziness.

Table 8 indicates significant associations between oral diabetic medications and post-coital hypoglycemia. The use of metformin (χ²=26.3, p<0.001), sitagliptin (χ²=13.1, p<0.001), gliclazide-sulfonylurea (χ²=10.7, p<0.001), and empagliflozin-sodium-glucose co-transporter 2 (SGLT2) (χ²=8.7, p=0.013) was significantly related to increased post-coital hypoglycemic events.

**Table 8:** Association Between Oral Diabetic Medications and Hypoglycemia During or After Coitus (Data presented as N (%); Chi-square test applied; p < 0.05 considered statistically significant) SGLT2: sodium-glucose co-transporter 2, GLP-1: glucagon-like peptide-1

Medication	Response	Hypoglycemia Yes (n, %)	Hypoglycemia No (n, %)	Not Applicable (n, %)	χ²	p-value
Metformin	Yes	15 (10.2%)	293 (57.0%)	112 (32.7%)	39.2	<0.001
Sitagliptin	Yes	5 (3.4%)	82 (13.8%)	43 (7.0%)	21.5	<0.001
Gliclazide-Sulfonylurea	Yes	2 (1.4%)	69 (11.3%)	29 (4.6%)	20.8	<0.001
Empagliflozin-SGLT-2	Yes	4 (2.7%)	35 (5.8%)	18 (2.8%)	6.2	0.013
GLP-1	Yes	1 (0.7%)	11 (1.8%)	8 (1.2%)	4.3	0.038

## Discussion

This study provides important insights into the prevalence and predictors of post-coital hypoglycemia among patients with T1DM and T2DM in Al-Ahsa, Saudi Arabia. We found that nearly one in five participants (17.9%) reported hypoglycemia during or after sexual activity. This prevalence underscores the significance of a complication that has received little clinical attention, despite its potential to affect quality of life and sexual health [11,12].

Our findings demonstrated that younger participants and those with higher educational attainment were more likely to report post-coital hypoglycemia. Age-related variation may be related to higher rates of sexual activity, greater physical exertion, and differences in insulin sensitivity among younger adults [13,14]. Education may also influence self-awareness and recognition of hypoglycemia symptoms, contributing to higher reporting [15]. Marital status emerged as another factor, with married individuals at significantly higher risk, likely reflecting more frequent sexual activity [16,17].

Insulin use was the strongest predictor of post-coital hypoglycemia, with nearly one-third of insulin users affected compared to only 3% of non-users. This supports previous evidence that intensive insulin therapy, while essential for glycemic control, increases vulnerability to exercise-related hypoglycemia [18,19]. Physiologically, sexual activity shares features with moderate-intensity exercise, with energy expenditure ranging from three to six metabolic equivalents and involving both sympathetic activation during arousal and parasympathetic dominance in the recovery phase [13,14,20]. These autonomic shifts may amplify glucose uptake in skeletal muscle and alter hepatic glucose output, predisposing insulin-treated patients to post-coital hypoglycemia [21,22].

Interestingly, our study also identified associations between post-coital hypoglycemia and the use of oral agents such as metformin, sitagliptin, gliclazide, and SGLT2 inhibitors. However, these medications have low intrinsic risk of hypoglycemia when prescribed as monotherapy [23,24]. The associations are more likely explained by confounding from combination therapy with insulin or sulfonylureas, which are well known to lower glucose excessively in susceptible patients [25,26]. We have therefore interpreted these results cautiously and emphasized the need for future studies to stratify exposures by treatment regimen.

Symptoms reported during post-coital hypoglycemia, including sweating, palpitations, dizziness, confusion, and hunger, were consistent with prior literature on autonomic and neuroglycopenic manifestations of hypoglycemia [12,27]. However, comorbid conditions such as hypertension, and the use of beta-blockers in particular, may blunt adrenergic warning signs and complicate recognition, thereby delaying treatment [28]. These findings highlight the importance of tailored patient education that considers both diabetes regimen and comorbidity profile.

Cultural context also warrants discussion. Sexual health remains a sensitive topic in many regions, including Saudi Arabia, and reliance on telephone interviews may have contributed to underreporting due to social desirability bias. Although assurances of confidentiality were provided, this methodological constraint must be acknowledged [29]. Furthermore, the cross-sectional design and single-region setting limit causal inference and generalizability. Nevertheless, the large sample size and inclusion of both T1DM and T2DM patients represent important strengths.

The clinical implications of this study are substantial. Healthcare providers should integrate discussions of sexual health into routine diabetes counseling and proactively address the risk of post-coital hypoglycemia, particularly for insulin-treated and younger patients. Practical strategies include monitoring blood glucose before and after intercourse, adjusting insulin dosage or timing, and consuming a small carbohydrate snack beforehand [30-32]. Partner education is also critical, as prompt recognition and assistance during hypoglycemic events can prevent severe outcomes [33].

Future research should aim to validate these findings using prospective designs and continuous glucose monitoring (CGM) to capture real-time glycemic fluctuations during and after sexual activity. Such approaches would provide more objective evidence, clarify mechanisms, and guide the development of targeted prevention strategies [34-36]. Multicenter studies across diverse cultural settings are also needed to enhance generalizability and to inform culturally sensitive patient education programs.

In summary, this study highlights that post-coital hypoglycemia is a prevalent and under-recognized complication in diabetes, driven primarily by insulin use and T1DM status, but also influenced by demographic and behavioral factors. By raising awareness and incorporating counseling into standard care, healthcare providers can help mitigate this risk, thereby improving both metabolic and sexual health outcomes in patients with diabetes.

## Conclusions

This study demonstrates that post-coital hypoglycemia is a significant yet under-recognized complication among patients with diabetes in Al-Ahsa, affecting nearly one in five participants, with the highest risk observed in insulin users and those with T1DM. Younger age, higher education, and marital status were also associated with greater risk, underscoring the interplay of clinical and behavioral factors.

The findings highlight the need for targeted patient counseling. Healthcare providers should incorporate discussions of sexual health into diabetes education, emphasizing practical strategies such as monitoring blood glucose before and after intercourse, consuming a small carbohydrate snack when appropriate, and adjusting insulin dose or timing to reduce hypoglycemia risk. Patients and their partners should also be educated about the recognition and management of hypoglycemia during sexual activity.

While the results provide novel insights, they represent associations rather than causal relationships due to the cross-sectional design. Cultural sensitivity and reliance on self-reported data may have contributed to underreporting. Future prospective studies, particularly those employing continuous glucose monitoring, are needed to validate these findings, clarify underlying mechanisms, and inform the development of culturally appropriate prevention strategies.

By raising awareness of post-coital hypoglycemia and integrating risk reduction into routine diabetes care, healthcare providers can improve not only metabolic outcomes but also the overall quality of life and sexual well-being of individuals with diabetes.
